# Immunothrombotic Activity of Damage-Associated Molecular Patterns and Extracellular Vesicles in Secondary Organ Failure Induced by Trauma and Sterile Insults

**DOI:** 10.3389/fimmu.2018.00190

**Published:** 2018-02-08

**Authors:** John Eppensteiner, Robert Patrick Davis, Andrew S. Barbas, Jean Kwun, Jaewoo Lee

**Affiliations:** ^1^Department of Surgery, Duke University, Durham, NC, United States

**Keywords:** damage-associated molecular pattern, extracellular vesicle, thrombosis, inflammation, polymer, trauma, cancer, transplantation

## Abstract

Despite significant improvements in injury prevention and emergency response, injury-related death and morbidity continues to increase in the US and worldwide. Patients with trauma, invasive operations, anti-cancer treatment, and organ transplantation produce a host of danger signals and high levels of pro-inflammatory and pro-thrombotic mediators, such as damage-associated molecular patterns (DAMPs) and extracellular vesicles (EVs). DAMPs (e.g., nucleic acids, histone, high-mobility group box 1 protein, and S100) are molecules released from injured, stressed, or activated cells that act as endogenous ligands of innate immune receptors, whereas EVs (e.g., microparticle and exosome) are membranous vesicles budding off from plasma membranes and act as messengers between cells. DAMPs and EVs can stimulate multiple innate immune signaling pathways and coagulation cascades, and uncontrolled DAMP and EV production causes systemic inflammatory and thrombotic complications and secondary organ failure (SOF). Thus, DAMPs and EVs represent potential therapeutic targets and diagnostic biomarkers for SOF. High plasma levels of DAMPs and EVs have been positively correlated with mortality and morbidity of patients or animals with trauma or surgical insults. Blocking or neutralizing DAMPs using antibodies or small molecules has been demonstrated to ameliorate sepsis and SOF in animal models. Furthermore, a membrane immobilized with nucleic acid-binding polymers captured and removed multiple DAMPs and EVs from extracellular fluids, thereby preventing the onset of DAMP- and EV-induced inflammatory and thrombotic complications *in vitro* and *in vivo*. In this review, we will summarize the current state of knowledge of DAMPs, EVs, and SOF and discuss potential therapeutics and preventive intervention for organ failure secondary to trauma, surgery, anti-cancer therapy, and allogeneic transplantation.

## Key Concepts

- **Secondary Organ Failure:** Dysfunction or injury of organs remote from the primary injury site is often the sequelae of the host’s dysregulated immune response.
- **Immunothrombotic Agents:** Certain cellular components released from stressed, damaged, or dead cells can activate both innate immune receptors and coagulation cascades, leading to inflammatory response and blood coagulation, respectively.
- **Feed-Forward Loop:** In solid organ transplantation, graft injury from infiltrating inflammatory cells leads to further DAMP release, intensified inflammation, and exacerbation of graft injury.
- **Vicious Cycle of Injury and DAMP/EV Production:** Polytrauma or invasive surgery will produce circulating pro-inflammatory and pro-thrombotic mediators that cause microinjury and *de novo* release of the pro-inflammatory and pro-thrombotic mediators in remote organs, thereby developing SOF.

## Introduction

About five million people die from injuries worldwide every year (1). Most injury deaths are immediate or early death, occurring within 2–3 days as a result of primary injuries, while 10–20% of injury deaths occur in the late phase (2). Secondary damage in organs remote from the primary site of injury causes 50–60% of late injury deaths (3). Secondary organ failure (SOF) is often caused by systemic, overwhelming inflammatory response following hemorrhage and reperfusion injury (3). Although SOF is most prevalent in patients with traumatic injuries, SOF also occurs in patients with sterile insults such as invasive surgery or anti-cancer treatment (4, 5). Injuries induce significant immune and thrombotic consequences at local and remote organ sites, as well as systemic circulatory changes. After injury, tissues release various cellular components into the extracellular space or bloodstream. These components play a key role in hemostasis, repair of damaged tissue, and initiation of host immune response against infection (6, 7). On the other hand, they are directly and indirectly involved in the pathogenesis of systemic inflammatory and thrombotic complications that cause multiple organ failure (MOF) (8, 9).

Damage-associated molecular patterns (DAMPs) are a broad array of molecules or molecular complexes released from damaged, stressed, or activated cells. DAMPs are recognized by various innate immune receptors called pattern recognition receptors (PRRs), e.g., toll-like receptors (TLRs), C-type lectin receptors, nucleotide-binding oligomerization domain-like receptors, retinoic acid-inducible gene I-like receptors, and receptors for advanced glycation end products (RAGE), which are expressed on both immune and non-immune cells (10). Each PRR recognizes a particular molecular pattern presented in DAMPs (Table 1). Upon binding to DAMPs, PRRs trigger intracellular signaling cascades that lead to the expression of inflammation-associated genes that have pleiotropic effects on host immune defense and pathogeneses (11).

**Table 1:** Immunothrombotic activity of DAMPs released after trauma and sepsis.

DAMP	Molecular classification	PRR	Coagulation activity	Pathologic plasma levels	Reference
Formyl peptide	Mitochondrial protein	FPR1	Unknown	Unknown	(12)
nDNA	Nucleic acid	TLR9, AIM2	Inhibits plasmin-mediated fibrin degradation	181,303 kilogenome equivalents/L	(13–15)
mtDNA	Nucleic acid	TLR9	Activates intrinsic coagulation pathway	2–3 µg/mL	(12, 16)
Heparan sulfate	Glycosaminoglycan	TLR4	Activates antithrombin	180 ng/mL	(17–19)
Histone	Nuclear protein	TLR2, TLR4, TLR9, and NLRP3	Unknown	10–230 µg/mL	(20, 21)
HMGB1	Nuclear protein	TLR2, TLR4, TLR9, RAGE	Inhibits protein C, upregulates TF expression	57–526 ng/mL	(22, 23)
Hyaluronan	Glycosaminoglycan	TLR2, TLR4, and NLRP3	Unknown	Unknown	(24)
S100	Cytosolic protein	TLR2, TLR4, and RAGE	Promotes thrombus formation	Unknown	(25, 26)
Uric acid	Metabolic breakdown component of purine nucleotides	NLRP3	Unknown	Unknown	(27)

*DAMPs, damage-associated molecular patterns; PRR, pattern recognition receptor; FPR1, formyl-peptide receptor 1; nDNA, nuclear DNA; TLR, toll-like receptor; NLRP3, nucleotide-binding oligomerization domain, leucine rich repeat and pyrin domain containing 3; HMGB1, high-mobility group box 1 protein; RAGE, receptor for advanced glycation end products; TF, tissue factor*.

Extracellular vesicles (EVs) are small membranous vesicles released from cells. EVs contain various cellular contents, such as proteins, DNA, and RNA and represent their parental cells. Thus, EVs may play a fundamental role in the communication between cells (28). On the other hand, EVs carry various pro-inflammatory and pro-coagulative mediators [e.g., mitochondrial DNA (mtDNA), high-mobility group box 1 protein (HMGB1), heat shock protein (HSP), tissue factor (TF), and phosphatidylserine] that modulate inflammatory response and coagulation (29, 30). This review will focus on discussing the pro-inflammatory and pro-thrombotic DAMPs and EVs in the pathogenesis of SOFs after trauma, invasive therapy, and organ transplantation. This review will also introduce potential therapeutics and preventive approaches for SOF.

## Secondary Organ Failure

The concept of MOF [also called multiple organ dysfunction syndrome (MODS)] following surgical or traumatic insult dates back to 1970s as Tilney et al. first described the phenomena as sequential organ failure after ruptured abdominal aneurysms (31). Eiseman et al. first used MOF in 1977 to describe a clinical presentation after trauma-initiated hospitalization (32). The spectrum of physiological dysfunction prior to MOF was subsequently refined with the advent of systemic inflammatory response syndrome (SIRS) in 1990s (33), which provides a systematic way to identify the systemic hyperinflammatory state and potential reversibility, prior to overt organ failure. Regardless of the etiology of insult, MOF is ultimately attributed to the loss of homeostatic host immune function resulting in irreversible tissue and organ damage.

Multiple organ failure can be further classified as primary or secondary. Primary organ failure refers to organ dysfunction directly attributed to the principal insult. SOF, however, is not necessarily the direct result of traumatic tissue or organ injury, but rather the sequelae of the host’s dysregulated immune response, and it may not be appreciated until days after the primary insult. While early organ failure as a result of polytraumatic injury or invasive surgical intervention may be intuitive, the exact pathogenesis of SOF is less clear. Multiple studies have attempted to elucidate the multimodal distribution of SOF following traumatic or surgical insult. Deitch introduced the “gut hypothesis” in 1989, which hypothesized that translocation of bacteria across the intestinal mucosal barrier occurs more easily following trauma or hemorrhagic shock, leading to a condition that mirrors septicemia (34). Meakins proposed the “two-hit model,” purporting that an initial surgical or traumatic insult primes the immune system for a second hit (infection or surgical intervention) (35). This second insult propagates an exaggerated SIRS response, causing SOF.

Multiple organ failure is the leading cause of mortality in late death after traumatic injury (36, 37). However, because a singular definition does not exist for post-traumatic MOF, the variance in its epidemiologic impact, time course, and pattern remains broad. Two commonly used scoring systems are the Marshall MODS score (38) and the Denver postinjury MOF score (39). These clinical adjuncts attempt to further stratify risk and categorize critically ill patients by combining objective measures of continuous variables over multiple organ systems for a quantifiable threshold of MOF (Table 2). A recent paper aimed to examine the predictive properties of the Marshall MODS and Denver postinjury MOF score to better understand, synthesize, and prognosticate MOF in trauma patients (40). Hutchings et al. showed that the incidence of post-trauma MOF varies between 22.8 and 58.5% of patients, depending on which scoring system was used, as the Denver MOF score tends to be more stringent than the Marshall MODS in categorizing organ failure (40). Although the overall incidence of MOF has decreased with the improvement of prehospital and early inhospital resuscitative strategies, the rate of MOF-related morbidity and mortality has not significantly changed over time (37). It is clear that the impact of MOF remains significant, and further studies to clarify the complex pathologic interplay are needed.

**Table 2:** Multiple organ failure scoring system.

Organ system	Degree of dysfunction				
	Grade 0	Grade 1	Grade 2	Grade 3	Grade 4
**(A) Marshall multiple organ dysfunction score**					

**Pulmonary**PaO_2_/FiO_2_ ratio	>300	226–300	151–225	76–150	≤75
**Renal**Creatinine (μmol/L)	≤100	101–200	201–350	351–500	>500
**Hepatic**Total bilirubin (μmol/L)	≤20	21–60	61–120	121–240	>250
**Cardiac**Pressure-adjusted HR (PAR^a^)	≤10	10.1–15.0	15.1–20.0	20.1–30.0	>30
**Coagulation**Platelet count (×10^3^/mm^3^)	>120	81–120	51–80	21–50	≤20
**Central nervous system**Glasgow Coma Score	15	13–14	10–12	7–9	≤6

**(B) Denver postinjury multiple organ failure score**					

PulmonaryPaO_2_/FiO_2_ ratio	>250	250–200	200–100	<100	
RenalCreatinine (μmol/L)	<159	160–210	211–420	>420	
HepaticTotal bilirubin (μmol/L)	<34	34–68	69–137	>137	
CardiacInotropes	None	1 inotrope at small dose	1 inotrope at moderate dose OR > 1 inotrope at small dose	1 inotrope at high dose OR > 2 inotropes at moderate dose	

*^a^PAR = (heart rate/mean arterial pressure) × central venous pressure*.

## Immunothrombotic DAMPs and EVs

### Extracellular DNA

Elevated levels of extracellular DNA (exDNA) in the form of nuclear DNA (nDNA), mtDNA, or neutrophil extracellular trap (NETs) are often found in patients with sepsis (41, 42), traumatic injury (14), cancer (43), autoimmune disease (44), cardiopulmonary bypass surgery (45), and solid organ transplantation (46) and are correlated with morbidity and mortality of these patients. The exDNAs released from mammalian cells and bacteria are known as potent innate immune stimulators. The unique cellular location of nucleic acid-sensing PRR probably explains how nucleic acid-sensing PRR in immune cells can distinguish dangerous nucleic acids from safe counterparts (47, 48). Barton et al. demonstrated that a chimeric TLR9, engineered for expression on the cell surface, could be activated by self-DNA that could not stimulate wild-type TLR9 in the endosomal compartment (49). Thus, free DNA without delivery into endosomal compartments may not stimulate TLR9. Prikhodko et al. demonstrated that the levels of circulating mtDNA significantly increased in trauma patients compared to those in healthy volunteers, but purified mtDNA could not stimulate innate immune cells (50). These data suggest that the level of exDNA in the blood may be a suboptimal marker for human disease. Development of new approaches to detect circulating exDNA that actually activate innate immune cells would be beneficial.

In addition to pro-inflammatory activity, exDNA may have potent pro-thrombotic activity. A prospective cohort study demonstrated that elevated plasma DNA was detected in 19 of 23 patients with pulmonary embolism and in none of the 49 patients with other diagnoses (pneumonia, myocardial infarction, thrombophlebitis, or normal lung scans) (51). Moreover, the levels of plasma mtDNA and nDNA were much higher in patients with massive pulmonary embolism than in patients with submassive pulmonary embolism or healthy controls (52). Fuchs et al. demonstrated that NETs, a meshwork of DNA fibers comprising histones and antimicrobial proteins, stimulated thrombus formation *in vitro* and *in vivo*, and treatment with deoxyribonuclease (DNase) or anticoagulant heparin prevented NET-mediated thrombus formation (53). Thus, exDNA acts as potent immunothrombotic agents.

### High-Mobility Group Box 1 Protein

High-mobility group box 1 protein is a non-histone nuclear protein composed of two positively charged DNA-binding motifs and a C-terminal acidic tail (54). HMGB1 is known to be passively released from dead and dying cells or actively released from live cells (55, 56). Circulating HMGB1 was markedly elevated in patients after traumatic injuries (22), ischemic injuries (57), severe acute pancreatitis (58), organ transplantation (59, 60), and arthritis (61, 62). Elevated plasma HMGB1 was significantly correlated with poor clinical outcome of these patients. In innate immune response, HMGB1 acts as an endogenous ligand of TLRs 2, 4, 9 and RAGE (63, 64). The innate immune stimulatory activity of HMGB1 has been determined by the redox state of cysteine residues C23, C45, and C106 (65). A disulfide bond between C23 and C45 is required for HMGB1 to activate innate immune cells and produce inflammatory cytokines (65), while reduction of all cysteine residues makes HMGB1 a chemoattractant rather than a cytokine inducer (66). By contrast, oxidization of the cysteine residues using reactive oxygen species abrogated both activities (66). Therefore, the level of total HMGB1 may be proximal in the innate immune response and may not accurately reflect the complex clinical nature of patients with inflammatory complications, as well as relevant clinical inflammation downstream signaling.

High-mobility group box 1 protein is also a potent pro-coagulant. HMGB1 directly stimulated and recruited platelets through TLR4 and RAGE (67–69). In a rat model, combined administration of thrombin and HMGB1 resulted in excessive fibrin deposition in glomeruli, prolonged plasma clotting times, and increased mortality (23). Mice with HMGB1-deficient platelets exhibited increased bleeding times and reduced thrombus formation, platelet aggregation, inflammation, and organ damage during experimental trauma/hemorrhagic shock (67). In a mouse venous thromboembolism (VTE) model, disulfide HMGB1 played a critical role in the development of venous thrombosis through facilitation of RAGE-dependent NET formation and platelet activation (56). Thus, HMGB1 is an important pathogenic factor of inflammatory and thrombotic complications.

### Histone

Histone is a cationic nuclear protein that packages DNA into nucleosomes. Extracellular histones are found in three different forms (free, DNA-bound nucleosome, and a part of NET) in the blood of patients with sepsis, trauma, ischemia and reperfusion injury (IRI), and autoimmune disease (70). In mouse models of concanavalin A-induced inflammatory complication and toxin-induced liver injury, extracellular histones contributed to mortality after inflammatory and cellular injuries through TLRs 2 and 4 (71). Moreover, extracellular histones exacerbated IRI through TLR9-medicated cytotoxic effects in a mouse hepatic IRI model (72).

On the other hand, recombinant human histones H3 and H4 directly triggered thrombin generation *in vitro* in a platelet-dependent manner (73). Moreover, extracellular histones upregulated the expression of TF on endothelial cells and macrophages through TLRs 2 and 4 (74). Extracellular histones activated platelets to aggregate through fibrinogen-mediated cross-linking of platelet integrin αIIbβ3, leading to profound thrombocytopenia and tissue damage in mice (75). Treatment with heparin could prevent histone-mediated thrombocytopenia and tissue damage *in vivo* (75). Clinically, elevated levels of circulating histones and histone-DNA complexes were associated with the incidence of MOF, disseminated intravascular coagulation, cardiac injury, arrhythmia, and ventricular dysfunction in patients with sepsis (76, 77). Therefore, the extracellular histone also acts as an immunothrombotic agent.

### S100

S100 proteins are a family of intracellular low-molecular weight, calcium-binding proteins. At least 25 distinct S100 proteins have been identified, and each S100 protein exerts diverse cellular functions in cell proliferation, differentiation, migration, calcium homeostasis, inflammation, and cell death (78). The S100 proteins are known to be either passively released from damaged cells or actively secreted from activated cells, and they have been detected in various body fluids, such as serum, urine, sputum, cerebrospinal fluid and feces of patients with cancer, inflammatory and autoimmune disease, or cardiovascular complications (79).

Extracellular S100 proteins act as potent pro-inflammatory and pro-thrombotic mediators. S100A1 released from damaged cardiomyocytes during myocardial infarction triggers TLR4-dependent pro-inflammatory responses, leading to induction of myocardial damage (80). S100A8, S100A9, and S100A12 induced TLR4-mediated inflammatory cytokine production by human peripheral blood mononuclear cells (81). In contrast, S100A9 induced RAGE-dependent cell migration of human monocytes and leukocytes (81), and S100B mediates neuronal damages in a RAGE- and NF-κB-dependent manner (82). Furthermore, S100A8/A9 heterodimeric proteins released from neutrophils induced RAGE-dependent activation of hepatic Kupffer cells, leading to the development of inflammatory thrombocytosis and atherogenesis in diabetic mice (83). Platelet-secreted S100A9 and S100A8/A9 proteins facilitated thrombus formation and occlusive thrombosis in mice with carotid artery injury (25).

### IL-1α and IL-33

IL-1α and IL-33 are expressed in precursor form in the nucleus of various hematopoietic cells, and these nuclear proteins play important roles in regulation of gene expression (79). IL-1α and IL-33 do not contain a secretory signal peptide, and thus they are released into extracellular space through either non-canonical vesicular secretion pathway or passive necrotic release (79). Proteolytic cleavage is required for the release and pro-inflammatory activity of IL-1α and IL-33 (79). Extracellular IL-1α interacts with ubiquitously expressed IL-1 receptor-1 and IL-1 receptor accessory protein (IL-1RAcP) that activates downstream signaling proteins, such as myeloid differentiation primary response gene 88 (MyD88) and interleukin-1 receptor-activated protein kinase 4, and induces an inflammatory response (84). IL-1α has been considered a potential pathogenic factor involved in the development and progression of diabetes (85), inflammatory bowel disease (86), myocardial inflammation (87), and cancer (88). Active IL-33 binds to the heterodimeric plasma membrane receptor complex, consisting of ST2 and IL-1RAcP, inducing NF-κB and mitogen-activated protein kinase (MAPK) activation and Th2 maturation (89). Elevated IL-33 expression in lung tissue and blood was correlated with the severity of asthma (89) and chronic obstructive pulmonary disease (90). IL-33 upregulated TF expression on endothelial cells in ST2 and NF-κB-dependent manner, promoting arterial thrombus formation after plaque rupture (91).

### Extracellular Vesicles

Extracellular vesicles comprise various membranous particles that originate from different intracellular origins and have different sizes. Thus, different types of EVs have been isolated by differential ultracentrifugations (92). Organelle size ranges from 0.5 to 10 µm. Microparticles (MPs) range from 0.1 to 1 µm in diameter, while apoptotic bodies range from 0.5 to 4 µm in diameter; both of these originate from plasma membranes. Exosomes are smaller than 0.1 µm and originate from multivesicular bodies (MVBs). Different types of EVs are generated by different mechanisms of biogenesis (30). For example, exosomes form by inward budding of MVB membranes while MPs and apoptotic bodies are generated by outward budding of plasma membrane (30). The biogenesis of MPs requires cytoskeletal reformations, such as redistribution of phospholipids, repositioning of phosphatidylserine, and contraction of the actin–myosin machinery (93, 94). The release of exosomes requires sequential assembly of the endosomal sorting complex on the MVB membrane (95). Upon release, EVs transfer their cargo by multiple mechanisms, such as endocytosis, phagocytosis, micropinocytosis, and membrane fusion (96, 97).

Depending on their origin, EVs carry various immune modulators and pro-coagulants (29, 30). Dendritic cell (DC)-derived exosomes express major histocompatibility complex (MHC) I, MHC II, and costimulatory molecules, and they can induce antigen-specific T cell responses (98). Interestingly, exosomes released from organ donor-derived DCs presented alloantigen and activated alloreactive T cells (99, 100). Exosomes and MPs released from cancer cells contributed to the suppression of host immune surveillance, cancer progression and metastasis, and angiogenesis (101–103). Like cancer-derived EVs, EVs released from mesenchymal stem cells attenuated host inflammatory responses and facilitated tissue regeneration, and are therefore being developed as therapeutic agents to treat graft-versus-host disease (104), chronic kidney disease (105), and acute radiation injury (106). On the other hand, MPs express various pro-coagulants, such as phosphatidylserine and TF (107), that promote vascular thrombosis in cancer patients (108). Moreover, endothelial and circulating cells after sepsis-induced microvascular injury released pro-thrombotic MPs into circulation (109). Thus, EVs may play dual roles in tissue repair and damage.

## Immunothrombotic Factors in Post-Trauma Organ Failures

The biologic response to traumatic injury is a complex physiological phenomena that involves a host of inflammatory and thrombotic mediators including cytokines, chemokines, complement, oxygen free radicals, inflammatory cells (neutrophils, monocytes, and macrophages), and endothelial cells (110). Immediately after traumatic injury, the immune response is mounted in reaction to cellular stress and tissue damage. Matzinger first introduced the “Danger Theory” in 1994 to explain how endogenous mediators released from damaged tissues can stimulate the innate immune response and elicit a nearly identical exaggerated SIRS response to that of infectious insult (111). This relatively new perspective of the immunostimulatory effects of self-molecules in sterile inflammation of a trauma model has challenged researchers to identify and categorize specific DAMPs.

The library of known DAMPs and their respective immunostimulatory consequences is constantly evolving as researchers continue to elucidate the complex signaling pathways involved. The release of DAMPs following traumatic injury promotes local inflammation and tissue repair, though when left unchecked leads to systemically injurious effects. One of the earliest characterized and well-known DAMPs in post-traumatic MOF is HMGB1, whose pathogenic effects were first outlined in experimental sepsis models. HMGB1 was found in the extracellular space after IRI and hemorrhagic shock (112) and led to inflammatory responses and microthrombotic effects through TLR4 and RAGE (23, 26). Given the multitude of inflammatory and coagulopathic effects, not surprisingly, elevated plasma levels of HMGB1 in critically ill trauma patients have been shown to be a negative prognostic indicator early in the course of traumatic disease (113).

Mitochondrial DNA is a well-studied endogenous signal for systemic inflammatory response after traumatic injury. Originally postulated from endosymbiotic theory, mtDNA shares many evolutionarily conserved molecular motifs with bacterial DNA (114), and human mtDNA is mostly unmethylated similar to bacterial DNAs (115). More recently, Zhang et al. have demonstrated that not only are mtDNA levels markedly elevated in trauma patients but also that *in vivo* shock-induced cell damage stimulates neutrophils to produce cytokines and cause end-organ damage *via* TLR9 (12, 116). 20–30% of CpG DNA across the genome of mammalian somatic tissues is unmethylated and may act as potential ligands of TLR9 (117). However, pathologic roles of unmethylated nDNA and mtDNA are debated in trauma and sepsis. Elevated mtDNA levels in the blood are positively correlated with the mortality of patients with sepsis or SOF in the intensive care unit (118). Furthermore, trauma patients who developed SIRS or MOF had elevated levels of mtDNA in their blood compared to those who did not (119). By contrast, the level of circulating nDNA, but not mtDNA was profoundly elevated in patients immediately after trauma, and the elevated nDNA was associated with immune suppression in these patients (120).

Another important pro-inflammatory mediator typically found in the mitochondrial matrix is N-formyl peptide, a well-established leukocyte chemoattractant (121). When released from necrotic or damaged cells, these proteins have been shown to aid in chemotaxis of neutrophils to sites of sterile inflammation (122). In addition, circulating histones caused direct cytotoxicity to epithelial and endothelial tissues by altering membrane permeability, which was associated with the incidence of acute lung injury after severe trauma (20). While an in-depth characterization is beyond the scope of this review, other well-known DAMPs mediating inflammation and tissue injury following trauma include but are not limited to HSP, uric acid, adenosine triphosphate, hyaluronan, galectins, and thioredoxin.

## Organ Damage and Failure after Solid Organ Transplantation

Damage-associated molecular pattern-mediated processes are increasingly recognized as important drivers of pathophysiology in solid organ transplantation (123, 124). The IRI process inherent to solid organ transplantation produces significant cellular injury with the concomitant release of multiple DAMPs. In turn, DAMPs are robust activators of the innate immune system, inciting inflammatory and thrombotic cascades that contribute to graft injury. More recently, the role of DAMPs in the subsequent activation of the alloimmune response has become an area of active investigation. Below, we review key aspects of DAMP biology in solid organ transplantation.

Damage-associated molecular patterns bind to PRRs on leukocytes and endothelial cells to initiate intracellular signaling cascades that lead to activation of the transcription factor NF-κB and increased gene expression of inflammatory response elements, particularly inflammatory cytokines. This cytokine milieu generates a sterile inflammatory environment and promotes infiltration of graft tissues with neutrophils and macrophages. Graft injury from infiltrating inflammatory cells contributes to a feed-forward loop leading to further DAMP release, intensified inflammation, and exacerbation of graft injury (123, 124). The clinical manifestation of these cellular events is the development of early allograft dysfunction (125). While the clinical definition of early allograft dysfunction differs by organ type, the common phenotype is insufficient physiologic function of the transplanted organ. In kidney transplantation, the immediate consequences of graft dysfunction are less severe, given the availability of renal replacement therapy by dialysis. However, for liver, lung, and heart transplantation, post-transplant graft dysfunction can be life-threatening.

Inflammation and thrombosis have a strong link, with significant interplay between elements of the inflammatory and coagulation cascades. Extracellular nucleic acid DAMPs (RNA and DNA) activate factors XII and XI in the coagulation cascade, inducing a pro-thrombotic state (126). The observation that such a pro-thrombotic state can be reversed through the actions of nucleic acid scavengers further supports the mechanistic link between DAMPs and thrombosis (127). The development of a pro-thrombotic state following solid organ transplantation has important clinical implications. One of the most severe complications following solid organ transplantation is the development of graft thrombosis. While important technical factors contribute to this complication (size and quality of blood vessels, surgical technique, etc.), there is growing recognition that the pro-thrombotic milieu generated by DAMP signaling may be an important risk factor warranting further investigation.

While DAMPs have been viewed predominantly as activators of the innate immune system, there is growing recognition that DAMPs represent an important link between the innate and adaptive immune response following solid organ transplantation (128). The initial inflammatory response generated by DAMP signaling leads to infiltration of the graft with host immune cells, including DCs and macrophages. These host antigen-presenting cells then traffic to local lymph nodes, presenting graft antigens to host T cells and initiating the adaptive immune response. Additionally, DAMP-mediated signaling potentiates the alloimmune response *via* increased expression of costimulatory and MHC molecules on antigen-presenting cells (129).

The clinical consequence of this array of molecular events is the development of acute allograft rejection. Allograft rejection is broadly categorized as either acute cellular rejection (T cell-mediated process) or antibody-mediated rejection (B cell-mediated process). The frequency and severity of rejection episodes vary greatly by organ type and by characteristics of the individual recipient. While acute rejection is usually reversible with high-dose steroid treatment, the clinical management of these complex patients continues to be a significant challenge routinely encountered by transplant physicians. Perhaps, an even more vexing and unsolved clinical problem in transplantation is the development of chronic allograft rejection. Chronic rejection is characterized by an inexorable decline in graft function over months or years, ultimately resulting in complete graft loss. Although chronic rejection manifests differently by organ type, this poorly understood process is frequently characterized by the development of graft fibrosis. Growing evidence demonstrates that DAMP signaling in response to subclinical immune injury over a prolonged time frame may contribute to the development of graft fibrosis (129). This exciting hypothesis warrants further study and may yield significant progress in combating one of the major clinical problems still limiting solid organ transplantation.

## DAMPs and EVs: Cancer Therapy-Induced Favorable and Adverse Effects

Thromboembolism is the obstruction of a blood vessel by abnormal clot formation in the circulation and a common fatal disease. Interestingly, VTE was 22-fold higher in patients with recent surgery, more than 12-fold higher in patients with recent trauma, 4.1-fold higher in patients with cancer, and 6.5-fold higher in patients undergoing anticancer therapies compared with healthy people (130). It is still unclear why VTE incidence elevates in such patients. Moreover, a precise prediction marker of VTE is indecisive. A growing body of evidence has demonstrated that increased levels of cancer-released MPs in the blood are highly correlated with the incidence of VTE in various types of cancers, e.g., malignant melanoma (131), pancreatic cancer (132), breast cancer (133), and glioblastoma (134). Furthermore, the level of transmembrane coagulation initiator TF in the blood was positively correlated with the recurrence of VTE in cancer patients (132, 135). Anticancer therapies increased the release of MPs from cancer cells (136–139) and upregulated the expression of TF on malignant and non-malignant cells (140, 141).

Depending on its mode of action, certain anticancer therapies, such as anthracycline chemotherapy (142, 143), radiation therapy (142, 144), transfection with PRR agonist polyriboinosinic:polyribocytidylic acid (145, 146), oncolytic virotherapy (147), and focused ultrasound ablation therapy (148), can induce substantial T cell-mediated antitumor responses. Unlike other cancer therapies, these anticancer therapies are known to induce immunogenic cancer cell death characterized by the release of tumor antigens and high levels of immune stimulators (DAMPs, adenosine triphosphate and cell-surface calreticulin) (142, 144, 149–151). DAMPs activate innate and adaptive immune cells *via* TLR and PRR signaling (152) while adenosine triphosphate and calreticulin act as “find-me” and “eat-me” signals, respectively, to recruit DCs and promote uptake and clearance of dead cells (153, 154). These DAMPs, find-me/eat-me signals, and tumor antigens cooperate to induce adaptive antitumor immune responses after treatment with immunogenic cancer cell death-inducing anticancer agents (155). On the other hand, uncontrolled DAMPs released from cancer cells with antineoplastic treatments are known as a risk factor of SOF in cancer patients (156–159). Therefore, it is evident that DAMPs and EVs released from cancer cells treated with anticancer therapeutic agents are double-edged swords in cancer therapy. Further studies are needed to elucidate mechanisms by which DAMPs and EVs produced by anticancer therapies contribute to the development of adverse and favorable responses in cancer patients.

## Therapeutics Targeting Uncontrolled DAMPs, EVs, and Downstream Signals

### TLR and TLR Signaling Inhibitors

#### Small Molecules

Eritoran, a synthetic lipid A antagonist, binds to and blocks the TLR4–MD2 complex. Intravenous administration of eritoran decreased lung injury and pulmonary inflammation and increased survival of mice infected with influenza (160). However, treatments with eritoran did not reduce mortality in patients with sepsis in a randomized, double-blind phase 3 clinical trial (161). 2-Acetamidopyranoside (C34) is a small molecule that tightly binds to the hydrophobic internal pocket of the TLR4–MD2 complex (162). C34 inhibited endotoxin-simulated TLR4 in enterocytes and macrophages *in vitro* and reduced systemic inflammation in the mouse models of endotoxemia and enterocolitis (162). Furthermore, C34 protected mice against acute lung injury after trauma/hemorrhagic shock (163). C34 has not been studied in humans.

TAK-242 is a small molecule TLR4 signaling inhibitor. TAK-242 as monotherapy failed to significantly reduce systemic inflammatory response, organ dysfunction, or survival in patients with sepsis, shock, or respiratory failure compared to a placebo control (164). TLR signaling pathways involve multiple transcription factors, e.g., NF-κB, c-Jun N-terminal kinase, and p38 MAPK. Inhibition of MAPK using a small molecule FR167653 suppressed lung heme oxygenase 1 expression and attenuated acute lung injury after hemorrhagic shock in mice (165). Furthermore, postburn treatments with MAPK inhibitor SB203580 prevented cardiac dysfunction after burn injury and resuscitation in rats (166). Interestingly, the SB203580 improved cardiac function but worsened lung injury and overall survival of mice with *Escherichia coli*-induced pneumonia, probably because of reduced innate immune response and bacteria clearance (167).

#### Anti-TLR Antibodies

Systemic blockade of TLR2 or TLR4 using monoclonal antibodies reduced systemic and pulmonary inflammation and mortality after polymicrobial sepsis in mice with cecal ligation and puncture (168). Furthermore, treatment with anti-TLR3 antibody attenuated ischemic gut injury and SOFs in mice with cecal ligation without puncture (169). A humanized anti-TLR2 antibody, OPN-305, reduced infarct size and myocardial necrosis and improved cardiac function in pigs after IRI (170).

#### Endosomal Acidification Inhibitor

Chloroquine and its derivative hydroxychloroquine are the prototype antimalaria drug. They also act as an endosomal acidification inhibitor, thus inhibiting endosomal TLRs 3, 7, and 9 (171). Administration of chloroquine after cecal ligation and puncture attenuated sepsis-induced MOFs and mortality in mice (172). In a rat hepatic IRI model, chloroquine treatment ameliorated acute liver injury at the early phase (0–6 h after reperfusion) but worsened liver injury at the late phase of reperfusion (24–48 h after reperfusion) (173). The mechanism of early protective action of chloroquine involved the modulation of MAPK activation and HMGB1 release, whereas chloroquine inhibited autophagy and induced hepatic apoptosis at the late phase (173). In addition to injury prevention, chloroquine and hydroxychloroquine have been used as anticancer therapeutic agents (174) and immunosuppressive agent for rheumatic diseases, lupus erythematosus, skin diseases, and graft-versus-host disease after bone marrow transplantation (175).

#### Oligonucleotide

Small oligonucleotides that bind to and inhibit TLR9 protected mice against sepsis-induced death (176). HMGB1 is an endogenous ligand of TLR4 and RAGE. In addition, HMGB1 can bind to immune stimulatory nucleic acids and facilitate nucleic acid-mediated innate immune stimulation (177). Non-immunogenic oligonucleotides were screened to bind to HMGB1 with high affinity but did not activate TLR9, and treatment with these non-immunogenic oligonucleotides protected mice against endotoxin-induced septic shock (177).

### DAMP and EV Inhibitors

#### Anti-HMGB1 and DNase

Because of the interconnectedness and redundancy of TLR and PRR signaling, the inhibition of single TLR and PRR signal pathways may be ineffective in ameliorating disease progress. Targeting upstream of TLR and PRR signal pathways would be more therapeutically effective than targeting downstream. Treatment with anti-HMGB1 antibody protected mice against acute lung injury after fracture and hemorrhagic shock (163). DNase I inhibited NET formation, and treatment with DNase I protected hepatocytes from cell death after IRI and significantly reduced IRI-induced liver injury in mice (178). Furthermore, intrathecal injection of DNase I prevented pulmonary endothelial dysfunction in rats with ventilator-associated pneumonia (179).

#### EV Inhibitors

Circulating exosomes after septic shock induced a statistically significant decrease in *in vitro* myocardial contractility compared with normal exosomes (180). Furthermore, exosomes isolated from septic patients induced vascular dysfunction by inducing reactive oxygen species generation and endothelial cell apoptosis (181). GW4869 is a neutral sphingomyelinase inhibitor. GW4869 inhibits sphingolipid ceramide-dependent release of exosomes from MVBs (182). Treatment with GW4869 prior to endotoxin challenge or cecal ligation and puncture in mice significantly reduced the levels of circulating exosomes and diminished sepsis-induced cardiac inflammation, myocardial dysfunction, and mortality (183).

#### Cationic Polymer-Based DAMP and EV Scavengers

Cationic polymers have been broadly used as nucleic acid transfection or drug delivery agents over the last few decades (184, 185). We have demonstrated that certain nucleic acid-binding cationic polymers (NABPs), e.g., polyamidoamine dendrimer, hexadimethrine bromide, and β-cyclodextrin-containing polymer, neutralized the ability of free DNA, RNA, and inorganic polyphosphate to activate nucleic acid-sensing TLRs (TLRs 3, 7, 8, and 9) (186) and intrinsic coagulation cascade (127). These NABPs were systemically administered to prevent TLR-mediated lethal liver injury (186). Furthermore, NABP-immobilized membranes simultaneously inhibited nucleic acid and non-nucleic acid DAMPs released from dead or dying cells or circulating in the blood of trauma patients, thereby preventing DAMP-induced inflammation and occlusive thrombosis in mice (187). NABP-immobilized hemoperfusion has a potential use during extracorporeal membrane oxygenation, continuous veno-venous hemofiltration, and continuous renal replacement therapy in intensive care units. Removing pro-inflammatory and pro-coagulative mediators from circulation is an unmet need in the treatment of critically ill patients.

## Conclusion

**Figure 1:**
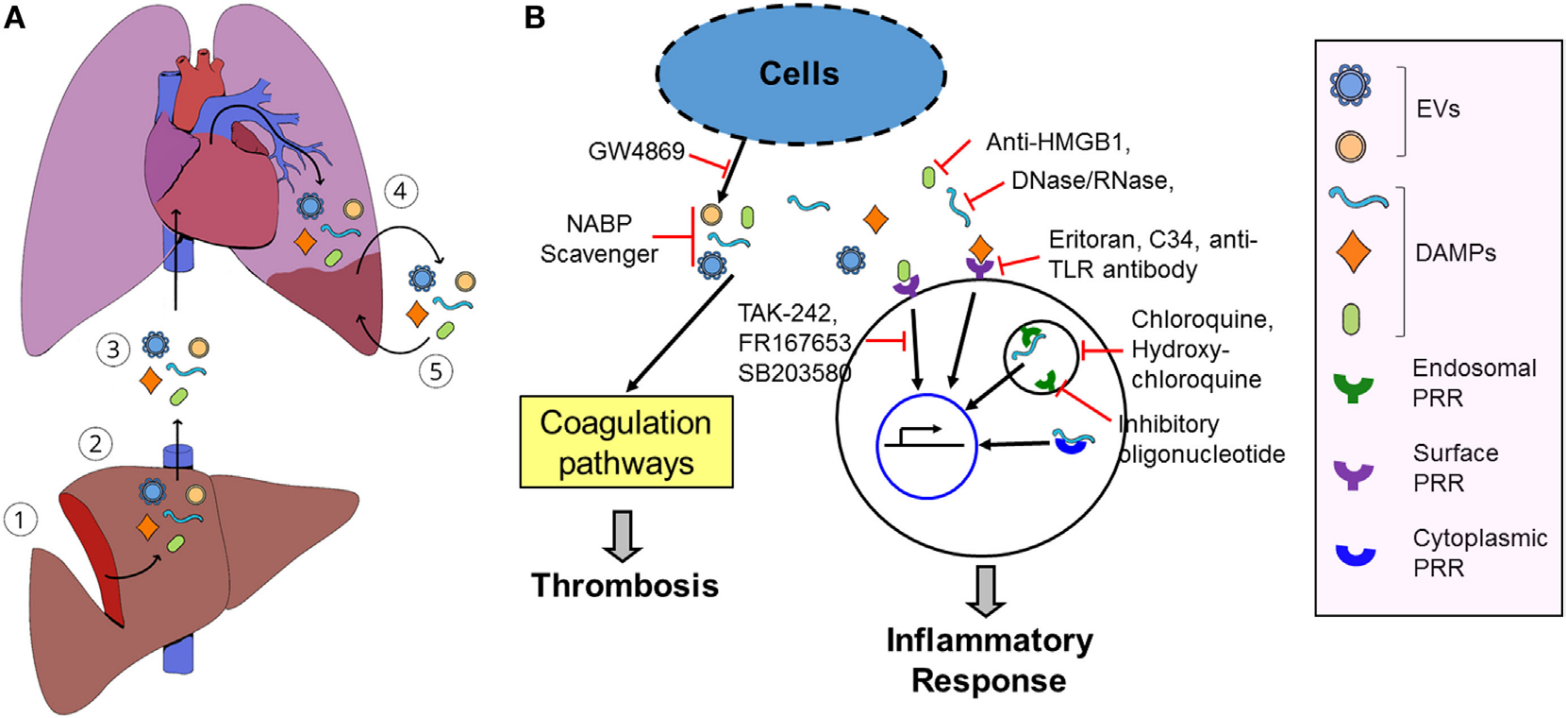
Model of damage-associated molecular pattern (DAMP)- and extracellular vesicle (EV)-induced secondary organ failure (SOF) and potential therapeutics. **(A)**, (1) Sterile insults cause primary tissue damage. (2) The damaged tissue releases various pro-inflammatory and pro-coagulative mediators, such as DAMPs and EVs. (3) Some mediators may not be cleared in the local damaged tissue and are released into the blood and circulated into remote organs. (4) These mediators will induce microthrombosis and local inflammation in the remote tissues, causing microinjuries. (5) The microinjured tissue subsequently releases *de novo* DAMPs and EVs, aggravating local tissue damages. Release of DAMPs and EVs and microinjuries in the remote tissues develops a vicious cycle and induces SOF. **(B)** Inhibition of inflammation and thrombosis using pattern recognition receptor (PRR) antagonists, PRR signaling inhibitor, DAMP inhibitor, EV biosynthesis inhibitors, and nucleic acid-binding cationic polymer (NABP) scavengers, thereby ameliorating and preventing SOF after tissue injury.

Patients with sterile insults produce a host of danger signals and high levels of pro-inflammatory and pro-thrombotic mediators after cellular injury and tissue damage. We postulate that circulating pro-inflammatory and pro-thrombotic mediators will cause the microinjury of organs remote from the primary site of injuries and *de novo* release of the pro-inflammatory and pro-thrombotic mediators, thereby developing a vicious cycle of the release of pro-coagulative and pro-inflammatory mediators and local tissue damage and subsequent development of SOFs (Figure 1). Breaking the vicious cycle will prevent SOF. Furthermore, pro-coagulative and pro-inflammatory mediators will develop as early prediction marker(s) for late-onset SOF in patients exposed to sterile insults.

## Author Contributions

Conceived and designed the concept: JL, JK, and AB. Wrote the manuscript: JL, JE, AB, JK, and RD.

## Conflict of Interest Statement

The authors declare that the research was conducted in the absence of any commercial or financial relationships that could be construed as a potential conflict of interest.

## Abbreviations

DAMP, damage-associated molecular pattern; DC, dendritic cell; DNase, deoxyribonuclease; EV, extracellular vesicle; exDNA, extracellular DNA; HMGB1, high-mobility group box 1 protein; IRI, ischemia and reperfusion injury; MAPK, mitogen-activated protein kinase; MHC, major histocompatibility complex; MODS, multiple organ dysfunction syndrome; MOF, multiple organ failure; mtDNA, mitochondrial DNA; NABPs, nucleic acid-binding cationic polymers; nDNA, nuclear DNA; NET, neutrophil extracellular trap; PRR, pattern recognition receptor; RAGE, receptor for advanced glycation end products; SIRS, systemic inflammatory response syndrome; SOF, secondary organ failure; TF, tissue factor; TLR, toll-like receptor; VTE, venous thromboembolism; FPR1, formyl-peptide receptor 1; AIM2, absent in melanoma 2; NLRP3, nucleotide-binding oligomerization domain, leucine rich repeat and pyrin domain containing 3.
